# Dietary intake, fatty acid in adipose tissue and cardio-metabolic health: cross-sectional study from the TwinsUK cohort

**DOI:** 10.1038/s41387-026-00438-6

**Published:** 2026-06-10

**Authors:** Xinyu Yan, Ricardo Costeira, Maryam Al-Hilal, Max Tomlinson, Panayiotis Louca, Emily R. Leeming, Cristina Menni, Thomas A. B. Sanders, Jordana T. Bell, Kerrin S. Small

**Affiliations:** 1 Department of Twin Research and Genetic Epidemiology, King’s College London, London, UK; 2 Health and Vital Statistics Division, National Center for Health Information, Ministry of Health, Sulaibkhat, Kuwait; 3 Department of Nutritional Sciences, King’s College London, London, UK; 4 Department of Pathophysiology and Transplantation, Università Degli Studi di Milano, Milan, Italy; 5 Fondazione IRCCS Cà Granda Ospedale Maggiore Policlinico, Angelo Bianchi Bonomi Hemophilia and Thrombosis Center, Milan, Italy

**Keywords:** Fat metabolism, Fatty acids, Cardiovascular diseases, Nutrition, Risk factors

## Abstract

**Background:**

Subcutaneous adipose tissue, the largest fat depot buffering fatty acids, correlates with cardio-metabolic diseases. Diet plays a major role in fatty acid metabolism and correlates with cardio-metabolic diseases. Studies have revealed clear links between plasma fatty acids with diet and cardio-metabolic diseases, but limited research focuses on fatty acids in subcutaneous adipose tissue.

**Methods:**

In TwinsUK, 18 subcutaneous adipose tissue fatty acid proportions were measured (*N* = 569) alongside dietary intake and clinical phenotypes. We investigated the association of adipose fatty acid levels and: (1) proxies of circadian factors; (2) eight dietary patterns; (3) cardio-metabolic risk phenotypes; and (4) the mediation effects of adipose fatty acids on the associations between diet and cardio-metabolic diseases.

**Results:**

(1) Proxies of circadian factors: We observed no association between adipose fatty acids and fasting hours or time of visit. (2) Dietary pattern: We identified associations between eight dietary patterns and adipose fatty acids, where the Healthy Diet Indicator showed the most associations. Most dietary scores positively correlated with polyunsaturated fatty acid (Beta = 0.10 [95% CI: 0.005, 0.19] − 0.23 [0.14, 0.32], *P*_FDR_ < 0.05), but Dietary Approaches to Stop Hypertension negatively correlated with two polyunsaturated fatty acids, arachidonic acid (Beta = −0.09 [−0.17,−0.006], *P*_*FDR*_ = 0.04) and adrenic acid (Beta = −0.09 [−0.18, −0.004], *P*_*FDR*_ = 0.04). Follow-up analysis showed that the intake of fat-rich food and fat-related nutrients were highly associated with fatty acid proportions. (3) Cardio-metabolic risk phenotypes including adiposity, lipids, glycemic and cardiovascular factors were associated with adipose fatty acids, where saturated and unsaturated fatty acids showed opposite directions. (4) Mediation effect: Adipose tissue proportion of arachidonic acid putatively mediated 24.7% of the total effect of Dietary Approaches to Stop Hypertension on visceral to gluteofemoral fat ratio.

**Conclusion:**

Adipose tissue fatty acid proportions represent dietary intakes and are associated with cardio-metabolic phenotypes. We provide a resource summarising the association between dietary measurements (dietary scores, nutrients and foods) and adipose tissue fatty acid proportions.

## Introduction

In 2021, non-communicable diseases accounted for 75% of deaths worldwide [1]. Cardiovascular diseases constituted the largest proportion of non-communicable diseases deaths, with an estimated 20.5 million fatalities in 2025, expected to almost double by 2050 [2]. Multiple studies have demonstrated that adherence to a healthy diet is significantly associated with reduced risk of major chronic diseases including cardiovascular diseases, type 2 diabetes, and cancer [3]. However, the underlying molecular mechanisms linking diet and health are poorly understood, though certain pathways such as inflammatory modulation [4], oxidative stress response [4], and gut microbiome interactions [5] have been partially elucidated.

Fatty acids are important metabolites, acting as energy suppliers, cellular membranes and signalling mediators [6]. Adipose tissue stores fatty acids, regulates a wide range of metabolic processes by secreting adipokines, cytokines, and pro- and anti-inflammatory proteins [7], and is implicated in the risk of cardio-metabolic diseases (CMD) [8]. Adipose fatty acids represent long-term dietary intake [9] and dietary intake is associated with CMD, highlighting a molecular bridge between what we eat and health outcomes. The relationships between dietary intake, serum fatty acid proportions, and CMD risk have been extensively studied over the past decades. For example, dietary intake of saturated fatty acids from foods high in animal fats is associated with increased risk of cardiovascular disease [10]. Dietary intake of polyunsaturated fatty acid (PUFA) is positively associated with n-3 and/or n-6 PUFA or total PUFA in serum or adipose tissue, but its link with CMD is subject to substantial controversy and uncertainty [11]. Research investigating the role of adipose tissue fatty acid composition in the association between dietary patterns and cardio-metabolic risk phenotypes is warranted.

Compared with using daily intake of food items or nutrients for research, a priori dietary patterns integrate cumulative effects of various dietary components to reflect the individual’s real-world habitual diet. One study reported strong links between serum metabolites and Mediterranean diet and CMD risk [12]. We sought to include eight commonly used dietary scores, such as Dietary Approaches to Stop Hypertension (DASH), Healthy Eating Index (HEI) and Healthy Diet Indicator (HDI). The DASH diet is rich in vegetables, fruits, and low-fat dairy foods, and with reduced saturated and total fat and is proposed by USDA to control blood pressure and prevent or treat hypertension [13]. The HEI is an index of overall diet quality developed according to USDA Food Guide Pyramid to both monitor population-level dietary changes over time and serve as a basis for nutrition promotion activities [14]. HDI is proposed based on World Health Organisation nutrition guidelines to prevent chronic diseases worldwide [15]. Given that each dietary score captures a slightly different aspect of diet and possesses distinct advantages and limitations, this study incorporates eight commonly reported dietary scores previously linked to CMD risk.

The circadian clock and feeding-fasting also play important roles in health [16]. Intermittent fasting and time-restricted diets are associated with lower-level risk biomarkers for cardio-metabolic disease and may increase health expectancy in humans [17], but frequent disruptions in circadian rhythm like shift work induce metabolic diseases [18]. Lipid patterns are intricately regulated by time of day and meal composition, and the postprandial responses vary between morning and afternoon [19]. Specifically, most circadian-oscillating metabolites are circulating fatty acids, peaking around midday [20]. Studies have shown extensive transcriptional response to fasting and a larger magnitude of the response to time of day in adipose tissue [21]. However, the relationship between circadian factors or their proxies, fasting and adipose tissue fatty acid proportions has not been well documented.

In this study, we profiled adipose fatty acids in female twins from a deeply phenotyped twin registry to (1) investigate the effect of time of day and fasting on adipose tissue fatty acids; (2) generate a resource of the association between three types of dietary measurements and adipose tissue fatty acids; (3) understand whether adipose tissue fatty acids mediate the effects of dietary patterns on cardio-metabolic risk phenotypes.

## Subjects and Methods

### Study population

TwinsUK is the largest adult twin registry in the UK developed to investigate the genetics and environmental factors of diseases with deep genotype and phenotype data [22]. Adipose tissue punch biopsies were collected from a subset of the TwinsUK cohort between 2007 and 2009, and fatty acid proportions were measured in 570 female twins of European ancestry (monozygotic twins (MZ) = 230, dizygotic twins (DZ) = 340). Biopsies were taken from a sunlight-protected area of the stomach adjacent and inferior to the umbilicus. On the day of biopsy collection, twins completed a half-day comprehensive clinical visit at which a range of phenotypes were collected, including anthropometry, whole-body scans, blood pressure, and blood samples were collected.

### Adipose fatty acid profiling

Fatty acid composition in subcutaneous adipose tissue was examined by fatty acid methyl esters (FAME), separated on an Agilent 7890 Gas Chromatograph (Agilent Technologies, California, United States) fitted with a 60 m capillary column BPX70 with hydrogen as carrier gas and a flame ionisation detector, as described by Christie [23]. The identities of fatty acids for which standards were unavailable were estimated using plots of the retention times to obtain the equivalent chain lengths and n-9/n-6/n-3 ratios, as described elsewhere [23]. Quality control was maintained by running a matrix standard of the sample of adipose tissue FAME with each run. Chromatograms were evaluated using ChemStation software (version 8.04) and the output was exported via a visual basic application into Excel for data analysis. Detailed information related to this process has been previously described [24]. Measures below the detection limit or failed quality control were set as NA. Altogether 569 female twins (MZ = 230, DZ = 339) passed the quality control. After quality assessment, 18 types of individual fatty acids were quantified as proportions to the total fatty acid. Fatty acid proportions were summed up to obtain the five fatty acid classes for each participant, saturated fatty acid (SFA), monounsaturated fatty acid (MUFA), and polyunsaturated fatty acid (PUFA) including n-3 PUFA and n-6 PUFA. Besides, the ratio of PUFA to SFA (PUFA/SFA) and the ratio of n-6 PUFA to n-3 PUFA (n-6 PUFA/n-3 PUFA) were also estimated. Adipose fatty acids (ratios) were rank-based inverse normalised before analyses.

### Assessment of fasting and time of day in sample collection

Hours fasting and time of day were investigated as exploratory proxies of circadian timing. To investigate the associations between hours fasting or time of day and adipose tissue fatty acids, we performed linear mixed-effects models (LMMs), adjusting for time of day or hours fasting, age, BMI, seasonality (day of year, transferred to sin- and cos-seasonality), and family relatedness. Twins came to clinical visits in working hours and were instructed to be fasted overnight. However, some twins did not fast overnight, so we further differentiated twins according to fasted overnight or not and considered this factor in the model.

### Dietary intake assessment

Habitual dietary intake was assessed using a 131-item food frequency questionnaire (FFQ), which has been previously validated against several biomarkers in the European Prospective Investigation into Diet and Cancer Norfolk (EPIC-Norfolk) study [25, 26]. The time interval between adipose fatty acid profiling and FFQ collection is within 0.98 (SD 0.30) years. FFQ EPIC Tool for Analysis (FETA) software was used to calculate the daily intake of nutrients and food groups [27]. Participants were excluded if there were ≥ 10 missing items or two standard deviations (SD) were outside the mean ratio of energy and basal metabolic rate [28]. A total of 427 participants (MZ = 172, DZ = 255) completed food frequency questionnaire and had adipose tissue fatty acids measures. Eight most frequently reported dietary scores against CMD risk were calculated, including Dietary Approaches to Stop Hypertension (DASH), Original Mediterranean Score (OMED), Amended Mediterranean Score (AMED), Diet Quality Index International (DQII), Healthy Eating Index (HEI), Alternative Healthy Eating Index (AHEI), Healthy Diet Indicator (HDI), and Nordic Dietary score (NDS) (Table S1), and details of calculation have been reported previously [29]. The agreement between dietary scores was measured using Pearson’s correlation (Fig. S1). The strongest correlation was observed between AMED and OMED (*r* = 0.86), whereas the weakest correlation was found between HEI and NDS (*r* = 0.28).

We also extended the diet fatty acid associations analyses to the nutrient and food intake level, which presented in more detail in the Supplementary material (Tables S2 and S3). Intake values of nutrients and food items were adjusted for energy intake using the residual method [30]. We also categorised 131 food items into 32 food groups defined by Hellbach et al. [31] (Table S4). Dietary intake were rank-based inverse normalised.

### Measures of cardio-metabolic risk phenotypes

This study analysed the following 14 cardio-metabolic risk phenotypes, which were all collected alongside the biopsy (Table S5 and Fig. S2): (1) Adiposity: body mass index (BMI), percentage of fat in the largest visceral fat region (Visceral fat%), and the ratio of percentage of fat in the largest visceral fat region to percentage of fat in gynoid region (VF%/GF%); (2) Lipids: high-density lipoproteins cholesterol (HDL-C), low-density lipoproteins cholesterol (LDL-C), total cholesterol, and total triglycerides; (3) Glycemic: fasting glucose, homeostasis model assessment of insulin resistance (HOMA2-IR), and fasting insulin; (4) Cardiovascular: systolic blood pressure (SBP), diastolic blood pressure (DBP), homocysteine, and Atherosclerotic Cardiovascular Disease (ASCVD) risk score. Cardio-metabolic risk phenotypes were rank-based inverse normalised before analyses. Age, physical activity, smoking and menopausal status were recorded as part of the self-report lifestyle questionnaire. Physical activity was assessed using the Index of Moderate (and) Vigorous Physical Activity which categorised participants into high (≥ 300), moderate (150 – 299), or low levels of activity (< 150) according to World Health Organisation guidelines.

### Statistical software and multiple testing adjustment

All statistical analysis was implemented using R version 3.6.2 [32]. Due to the multiple testing inherent in this high-dimensional analysis and inter-correlations among different adipose fatty acids, so as dietary patterns and cardio-metabolic risk indicators, we chose a more flexible method, Benjamini-Hochberg False Discovery Rate (FDR) at 0.05, to adjust *P* values unless stated otherwise. In contrast to Bonferroni correction, which is too conservative and stringent for exploratory analysis, severely reducing the power to find real effects and leading to many false negatives, FDR approach balances the identification of biologically relevant associations while limiting the proportion of false positive findings.

### Association tests

**Fig. 1:**
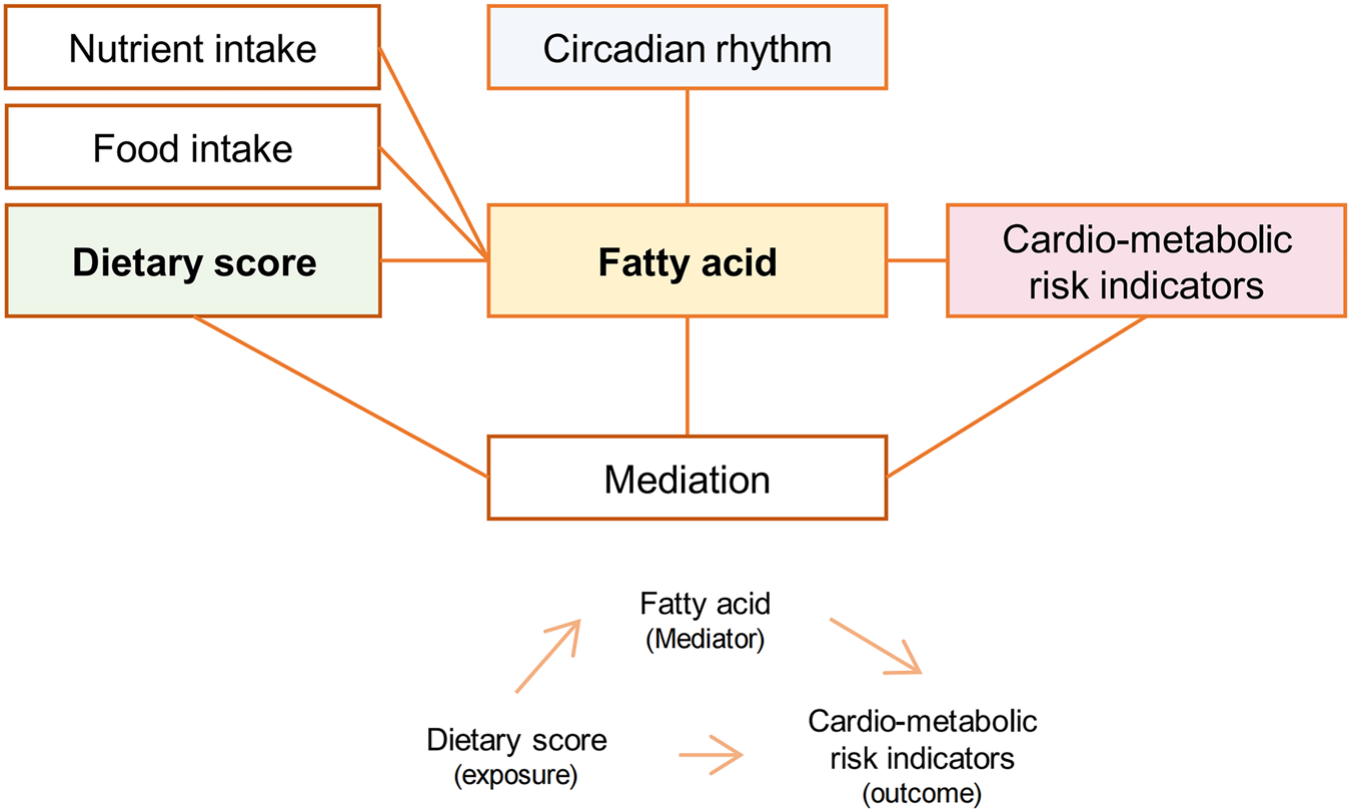
Overall study design. Graphical schematic of the study design. Graphical abstracts: summary of the study design.

The analyses were conducted in three stages. First, associations between dietary scores (or food group intake and nutrient intake in the Supplementary material) and fatty acid proportions in adipose tissue were tested with LMMs. In the LMMs, fatty acid proportions in adipose tissue were the response variables and dietary intakes were the predictors [33], adjusting for energy intake, alcohol intake, age, smoking status, physical activity, Index of Multiple Deprivation (IMD), and family relatedness (Model 1). To note, energy excluding energy from alcohol not total energy intake was included for the predictor HDI. Alcohol intake was included for the predictors A-HEI, HDI, DASH, and NDS as appropriate. The second stage included performing LMMs where dietary scores or adipose fatty acids were fixed effects predictors, and cardio-metabolic risk phenotypes were response variables adjusting for a set of covariates included in model 1. In the third stage, to test if the observed associations were robust, we added a series of covariates in the models testing the associations among dietary scores, adipose fatty acids and cardio-metabolic risk phenotypes. The study design was described in Fig. 1. In brief, these spanned inclusion of menopausal status, BMI, and cell types (adipocytes, macrophages, and microvascular endothelial cells) in sensitivity analyses.

Taking the association between adipose fatty acid and dietary scores as an example, the models we used for analysis were as follows:

$$\begin{array}{l}{\mathrm{Model}}\,1\,({\mathrm{Main}}\,{\mathrm{analysis}}):{\mathrm{Adipose}}\,{\mathrm{fatty}}\,{\mathrm{acid}}\sim{\mathrm{Dietary}}\,{\mathrm{score}}+\\{\mathrm{Energy}}\,(+\,{\mathrm{Alcohol}})+{\mathrm{Age}}+{\mathrm{Smoking}}\,{\mathrm{status}}+{\mathrm{Physical}}\,{\mathrm{activity}}\\ +\,{\mathrm{IMD}}+(1|{\mathrm{Family}})+(1|{\mathrm{Zygosity}})\end{array}$$

$$\begin{array}{l}\mathrm{Model\,}2\,(\mathrm{Sensitivity}\,\mathrm{analysis}):\mathrm{Adipose}\,\mathrm{fatty}\,\mathrm{acid} \sim \mathrm{Dietary}\\ \mathrm{score}+\mathrm{Energy\,}(+\mathrm{\,Alcohol})+\mathrm{Age}+\mathrm{Smoking}\,\mathrm{status}+\mathrm{Physical}\\ \,\mathrm{activity}+\mathrm{IMD}+\mathrm{Menopause}\,\mathrm{status}+(1|\mathrm{Family})+(1|\mathrm{Zygosity})\end{array}$$

$$\begin{array}{l}{\mathrm{Model}}\,3\,({\mathrm{Sensitivity}}\,{\mathrm{analysis}}):{\mathrm{Adipose}}\,{\mathrm{fatty}}\,{\mathrm{acid}} \sim{\mathrm{Dietary}}\\{\mathrm{score}}+{\mathrm{Energy}}\,(+\,{\mathrm{Alcohol}})+{\mathrm{Age}}+{\mathrm{Smoking}}\,{\mathrm{status}}+{\mathrm{Physical}}\\ \,{\mathrm{activity}}+{\mathrm{IMD}}+{\mathrm{BMI}}+(1|{\mathrm{Family}})+(1|{\mathrm{Zygosity}})\end{array}$$

$$\begin{array}{l}{\mathrm{Model}}\,4\,({\mathrm{Sensitivity}}\,{\mathrm{analysis}}):{\mathrm{Adipose}}\,{\mathrm{fatty}}\,{\mathrm{acid}} \sim{\mathrm{Dietary}}\\{\mathrm{score}}+{\mathrm{Energy}}\,(+\,{\mathrm{Alcohol}})+{\mathrm{Age}}+{\mathrm{Smoking}}\,{\mathrm{status}}+{\mathrm{Physical}}\\ \,{\mathrm{activity}}+{\mathrm{IMD}}+{\mathrm{BMI}}+{\mathrm{Adipocytes}}+{\mathrm{Macrophages}}+\\ \mathrm{Microvascular}\,\mathrm{endothelial}\,\mathrm{cells}+(1|\mathrm{Family})+(1|\mathrm{Zygosity})\end{array}$$

### Mediation analyses

We further investigated evidence for putative mediation effects of adipose fatty acids on the associations between dietary scores and cardio-metabolic risk phenotypes using R-package “mediation” [34] (Fig. 1). The proportion mediated shows the proportion of the effect of the independent variable on the dependent variable that goes through the mediator, calculated by dividing the average causal mediation (indirect) effects by the total (direct + indirect) effect. We selected significantly associated triplets (*P*_FDR_ < 0.05) across dietary scores, adipose fatty acids, and cardio-metabolic risk phenotypes, resulting in 16 independent tests (across three dietary scores, eight adipose fatty acids, and two cardio-metabolic risk phenotypes). Given the confirmatory nature of testing these specific putatively causal pathways and the strong implications of a mediation effect, a Bonferroni correction was applied to the mediation tests to stringently control the family-wise error rate. We reported both significant results with Bonferroni-corrected *P* < 0.05/16 and suggestive results with nominal *P* < 0.05.

## Results

### Study population characteristics

This study included 569 female twins with adipose tissue fatty acid data from the TwinsUK cohort [35], and analyses of dietary intake included the subset of 427 participants who had both adipose fatty acid and diet data (Table 1). The participants had an average age of 59 (SD 9) years and their average daily energy intake was 7870.5 (SD 2110.6) kJ (1880.2 (SD 504.2) kcal). More than half of the participants were engaged in low physical activity (89.9%) and were non-smokers (64.4%).

**Table 1:** Demographic characteristics of the study population.

Demographic Characteristics	Mean (SD) / N (%)	Missingness (%)
Age (years)	59 (9)	0
Smoking status		0.2
Never	236 (55.3)	
Current smoker	151 (35.4)	
Ex-smoker	39 (9.1)	
Physical activity level		0
Medium to high	43 (10.1)	
Low	384 (89.9)	
IMD (decile)	7.0 (2.4)	0
1	5 (1.2)	
2	15 (3.5)	
3	24 (5.6)	
4	33 (7.7)	
5	40 (9.4)	
6	48 (11.2)	
7	52 (12.2)	
8	50 (11.7)	
9	90 (21.1)	
10	70 (16.4)	
Menopausal status		12.4
Pre-menopausal	58 (13.6)	
Post-menopausal + Not on HRT	300 (70.3)	
Post-menopausal + On HRT	16 (3.7)	
BMI	26.1 (4.5)	0

The table shows mean and standard deviation (SD) for continuous variables, or sample size (N) and percentage (%) for categorical variables; The percentage of missingness is presented; HRT, hormone replacement therapy; IMD, index of multiple deprivation.

### Distribution of fatty acid content in adipose tissue

We measured the content of fatty acids in abdominal subcutaneous adipose tissue samples from 569 female twins. The study quantified 18 types of individual fatty acids, including six SFAs, three MUFAs, one trans-unsaturated fatty acid (TFA), and eight PUFAs including four n-3 PUFAs and four n-6 PUFAs (Table 2). In adipose tissue, C18:1n-9 (oleic acid) accounted for 46.8% of total fatty acids, followed by C16:0 (palmitic acid) 19.3%, C18:2n-6 (linoleic acid) 11.9% and C16:1n-7 (palmitoleic acid) 5.8%, presenting in similar proportions to those reported by Craig et al. [36] and Hodson et al. [37]. The majority of fatty acids are derived from the diet or by endogenous synthesis (de novo lipogenesis or remodelling of fatty acids, i.e., conversion of short- and medium-chain saturated fatty acids to palmitic acid) from excess energy intake. However, essential fatty acids, such as C18:2n-6 (linoleic acid) and C18:3n-3 (α-linolenic acid), must be obtained through the diet because humans lack the desaturase enzymes necessary for their synthesis [38, 39]. The proportions of the essential fatty acids varied, C18:2n-6 consisted of 11.9% while C18:3n-3 consisted of 0.9%. TFAs are not synthesised de novo and accumulate from partially hydrogenated vegetable oils and from fats derived from ruminants. TFA proportion was much lower than earlier reports from the UK [40] and reflects the removal of partially hydrogenated fats from the food chain. Overall, the fatty acid proportions in this study were similar to fatty acid proportions previously reported in adipose tissue [36, 37].

**Table 2:** Distribution of fatty acid content in adipose tissue.

Fatty Acids	Common Name	*N*	Median	Mean	SD
C10:0	Capric acid	546	0.25	0.33	0.36
C12:0	Lauric acid	566	0.34	0.61	0.73
C14:0	Myristic acid	567	2.17	2.03	0.87
C16:0	Palmitic acid	569	19.28	19.24	2.06
C18:0	Stearic acid	569	3.10	3.21	0.94
C20:0	Arachidic acid	563	0.35	0.36	0.17
SFA	Saturated fatty acid	543	25.62	25.74	2.88
C16:1n-7	Palmitoleic acid	567	5.75	5.79	1.66
C18:1n-7	Vaccenic acid	504	0.29	0.88	0.94
C18:1n-9	Oleic acid	569	46.77	46.55	3.05
MUFA	Monounsaturated fatty acid	502	53.25	52.97	3.27
C18:1trans	Elaidic acid, trans-vaccenic acid	497	0.51	0.53	0.30
TFA	trans-unsaturated fatty acid	497	0.51	0.53	0.30
C18:3n-3	α-Linolenic acid (ALA)	564	0.85	0.85	0.22
C20:5n-3	Eicosapentaenoic acid (EPA)	561	0.17	0.22	0.14
C22:5n-3	Docosapentaenoic acid (DPA)	561	0.36	0.39	0.17
C22:6n-3	Docosahexaenoic acid (DHA)	561	0.31	0.36	0.22
n-3 PUFA	n-3 (ω-3) polyunsaturated fatty acid	559	1.73	1.81	0.50
C18:2n-6	Linoleic acid (LA)	568	11.92	12.05	2.37
C20:3n-6	Dihomo-γ-linolenic acid (DGLA)	563	0.27	0.32	0.17
C20:4n-6	Arachidonic acid (AA)	563	0.54	0.56	0.17
C22:4n-6	Adrenic acid	561	0.21	0.26	0.17
n-6 PUFA	n-6 (ω-6) polyunsaturated fatty acid	558	13.04	13.22	2.26
PUFA	Polyunsaturated fatty acid	557	14.79	15.03	2.46
n-6 PUFA/n-3 PUFA	n-6 (ω-6) to n-3 (ω-3) PUFA ratio	557	7.60	7.72	2.19
PUFA/SFA	PUFA to SFA ratio	540	0.58	0.60	0.14

Median, mean and standard deviation (SD) showed the distribution of the proportion of each type of fatty acid in the subcutaneous adipose biopsy. N, sample size.

### Proxies of circadian rhythm and adipose fatty acid levels

**Fig. 2:**
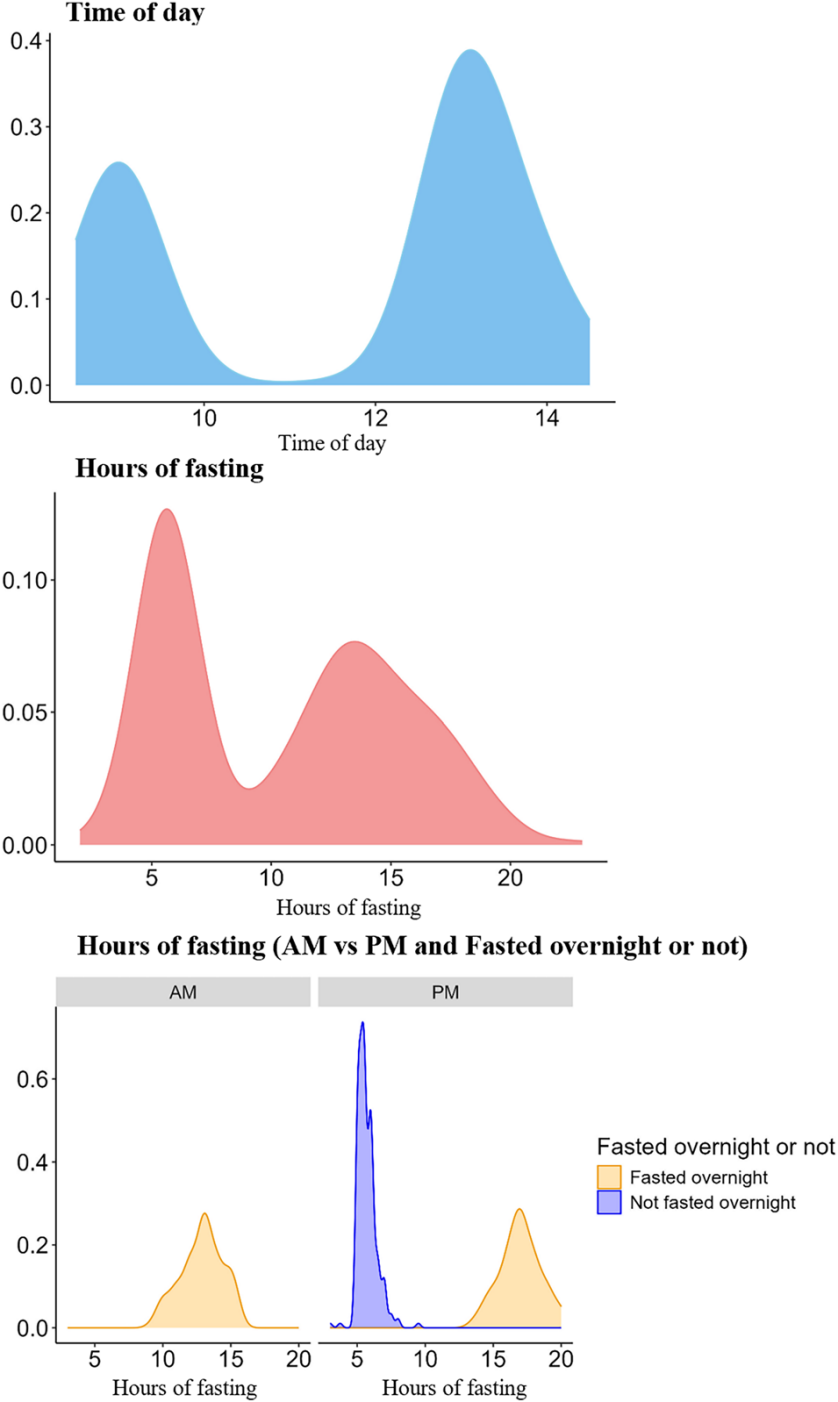
Time of day and hours of fasting at time of sample collection. The top plot displays the time of day that the biopsies were collected. The middle plot displays the hours of fasting the research volunteer had completed at time of biopsy collection. The bottom plot display the hours of fasting the research volunteer had completed divided into those that had fasted overnight and those that had not. The bottom left plot includes all biopsies collected in the AM (before midday) and the bottom right plot includes all biopsies collected in the PM (after midday).

To investigate the effects of circadian factors on adipose fatty acids, we utilised the proxy measures of recorded arrival time and fasted hours of participants on the clinical visit days, alongside adipose tissue biopsies collected at the same clinical visits. We included 498 participants who fasted, with most participants coming in the afternoon (*N* = 318) versus participants who came in the morning (*N* = 180) (Fig. 2). We first tested the associations between the time of the clinical visits and adipose fatty acids, but no significant result was identified (*P*_FDR_ > 0.05) (Table S6). Although participants were instructed to fast before the clinical visit, there was a large variation in the hours participants fasted. We found that no individual fatty acid was associated with hours of fasting (*P*_FDR_ > 0.05) (Table S6). In summary, adipose fatty acids were not significantly associated with proxies of circadian factors including time of day and hours of fasting. This is in marked contrast to serum, where circulating fatty acids oscillate with circadian factors including time of day [20]. In conclusion, unlike serum samples which tend to reflect recent intake, adipose tissue is a more robust marker of long-term intake.

### Dietary patterns and adipose fatty acids

**Fig. 3:**
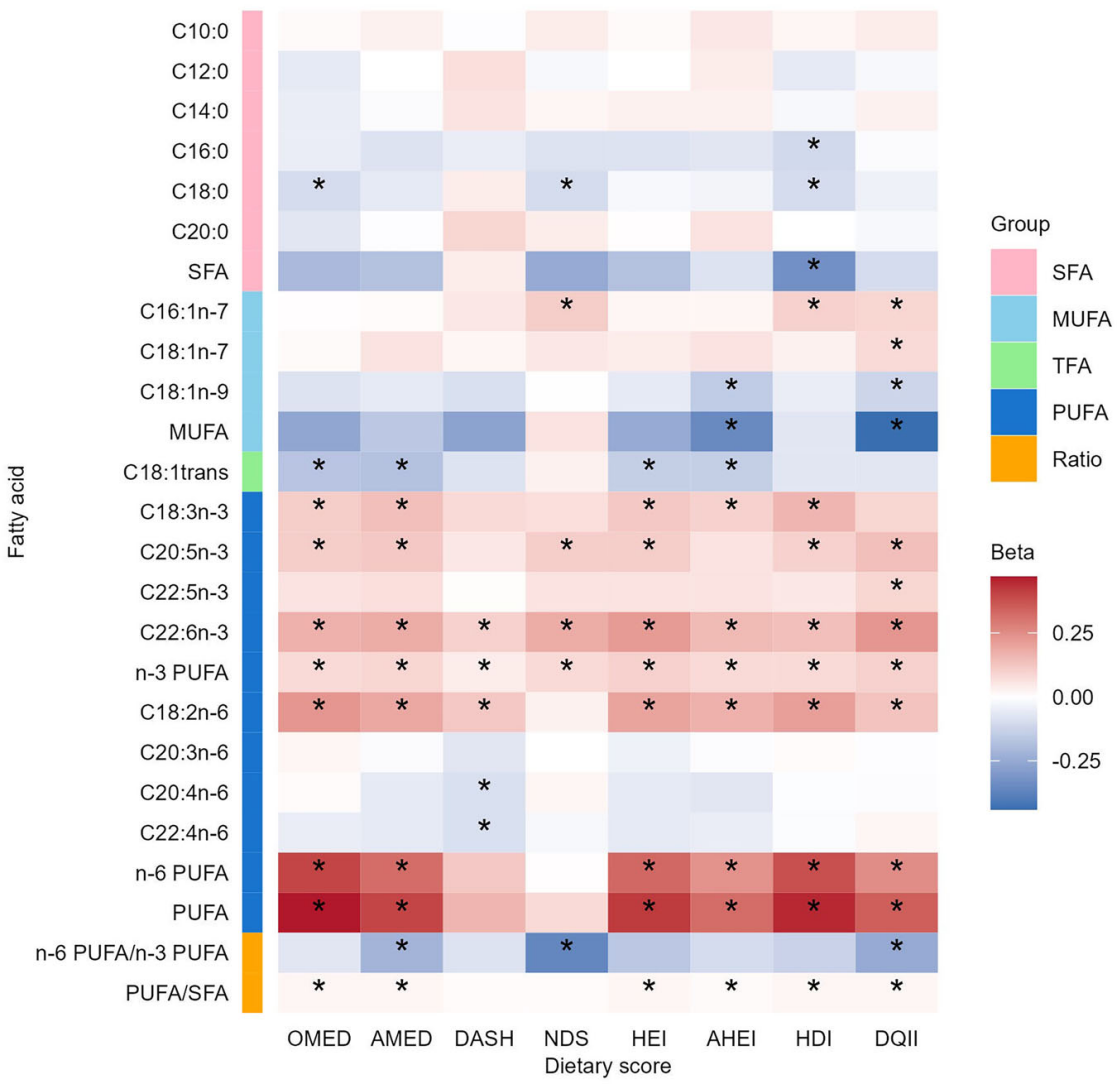
Associations between dietary scores and adipose fatty acids. The colour scale indicates the effect (Beta) of each dietary score on fatty acid levels. The asterisks show the significant (* *P*_FDR_ < 0.05). OMED, Original Mediterranean Score; AMED, Amended Mediterranean Score; DASH, Dietary Approaches to Stop Hypertension; NDS, Nordic Dietary score; HEI Healthy Eating Index, AHEI Alternative Healthy Eating Index, HDI Healthy Diet Indicator, DQII Diet Quality Index International, SFA saturated fatty acid, MUFA monounsaturated fatty acid, TFA trans-unsaturated fatty acid, PUFA polyunsaturated fatty acid.

To investigate the effects of dietary patterns on adipose fatty acids, we tested for the associations between eight dietary scores (DASH, OMED, AMED, DQII, HEI, AHEI, HDI, and NDS) and 25 fatty acid proportions or ratios (18 fatty acids, five summary values - SFA, MUFA, n-3 PUFA, n-6 PUFA and PUFA, and two ratios of fatty acid sums - PUFA/SFA and n-6 PUFA/n-3 PUFA), adjusting for age, smoking status, physical activity, IMD, and family relatedness. In brief, we observe the most associations between HDI and adipose fatty acids. The HDI score was negatively correlated with the proportions of two SFAs (C16:0 and C18:0, with coefficients [95% CI] -0.11 [-0.20 to -0.02] and -0.10 [-0.19 to -0.007], respectively, both *P*_FDR_ < 0.05) but was positively associated with the proportions of MUFA and PUFAs (C16:1n-7, C18:3n-3, C20:5n-3, C22:6n-3 and C18:2n-6, with coefficients [95% CI] ranging from 0.10 [0.01 to 0.19] to 0.21 [0.12 to 0.30], all *P*_FDR_ < 0.05) (Fig. 3 and Table S7). The highest numbers of associations was observed with PUFAs; All selected dietary scores were positively associated with adipose proportions of C18:3n-3 (α-linolenic acid), C20:5n-3 (eicosapentaenoic acid), C22:6n-3 (docosahexaenoic acid) and C18:2n-6 (linoleic acid), with coefficients [95% CI] ranging from 0.10 [0.005 to 0.19] to 0.23 [0.14 to 0.32] (all *P*_FDR_ < 0.05). The exception was the DASH score which was negatively correlated with another two types of PUFAs, C20:4n-6 (arachidonic acid) (coefficient [95% CI] −0.09 [−0.17 to −0.006], *P*_FDR_ = 0.04) and C22:4n-6 (adrenic acid) (coefficient [95% CI] −0.09 [−0.18 to −0.004], *P*_FDR_ = 0.04). A limited number of associations were identified between dietary scores and adipose proportions of SFAs and MUFAs; The AHEI and DQII scores were negatively correlated with the proportion of C18:1n-9 (oleic acid) (coefficient [95% CI]: −0.15 [−0.24 to −0.06] and −0.12 [−0.21 to −0.03], respectively, both *P*_FDR_ < 0.05). The Mediterranean Score (OMED and AMED) and Healthy Eating Index (HEI and AHEI) were negatively correlated with the proportion of C18:1trans (trans-vaccenic acid), with coefficients [95% CI] ranging from −0.18 [−0.27 to −0.09] to −0.14 [−0.24 to −0.04] (all *P*_FDR_ < 0.05). Collectively, adherence to HDI is the potential best dietary pattern to predict fatty acid distributions in adipose tissue. Adipose proportions of α-linolenic acid, eicosapentaenoic acid, docosahexaenoic acid and linoleic acid in adipose tissue are potential biomarkers of CMD-related dietary patterns.

**Fig. 4:**
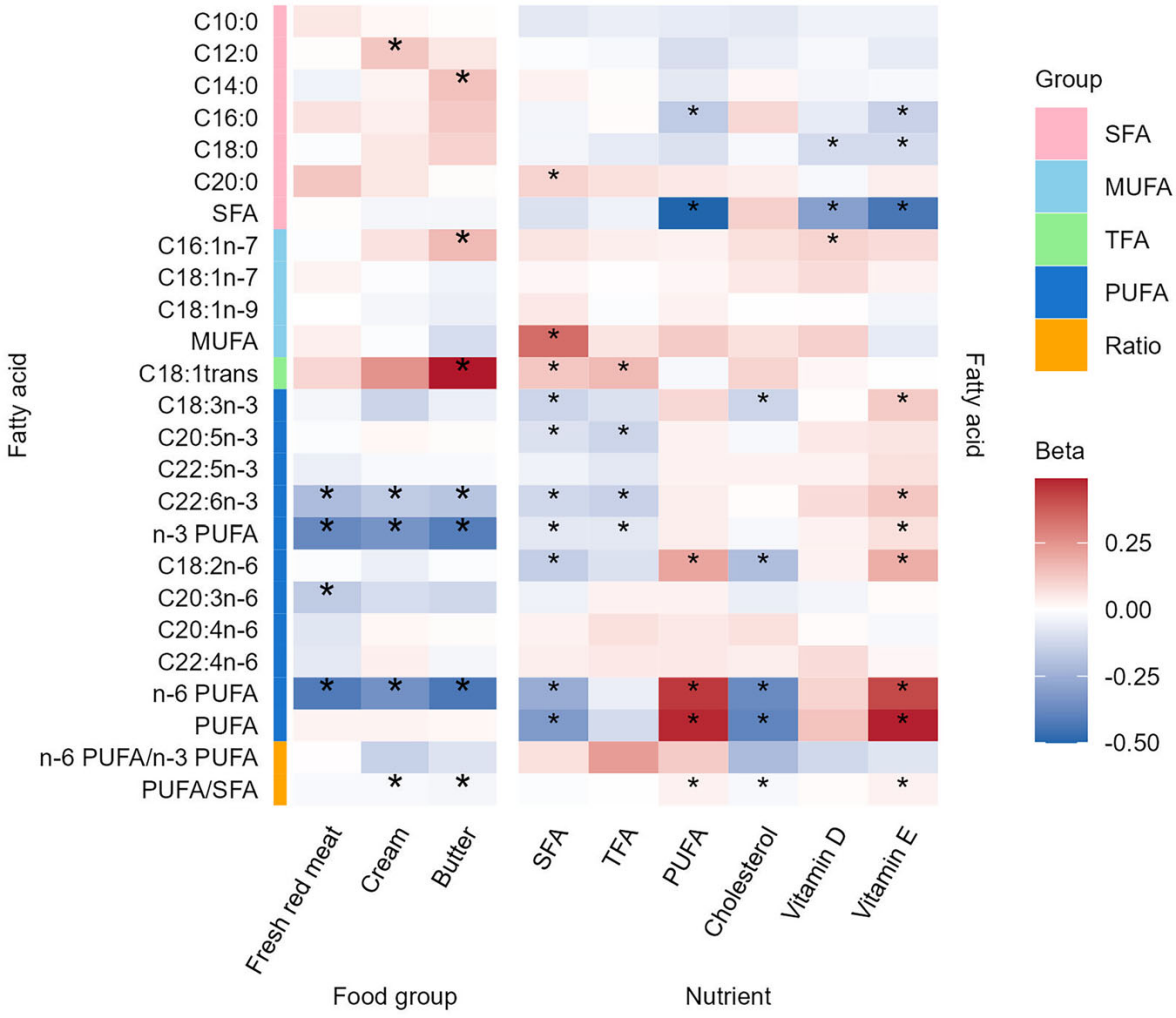
Associations between fat-rich food group and fat-related nutrient intake and adipose fatty acids. The colour scale indicates the effect (Beta) of each nutrient on fatty acid levels. The asterisks show the significant (* *P*_FDR_ < 0.05). SFA saturated fatty acid, MUFA monounsaturated fatty acid, TFA trans-unsaturated fatty acid, PUFA polyunsaturated fatty acid.

To examine whether certain food or nutrient intakes drive the dietary pattern associations, we tested the associations between nutrient and food intake and adipose fatty acids. Overall, as expected, the intake of fat-rich food and fat-related nutrients were highly associated with fatty acid proportions in adipose tissue (Fig. 4). We also observed negative associations between fresh red meat (including lamb and beef), butter, and cream intake and PUFA proportions in adipose tissue. We identify the expected positive association between polyunsaturated margarine and fish intake and PUFA proportions, as well as butter and cream intake and SFA proportions. In terms of nutrient intake, fat-related nutrient intake comprising PUFA, SFA and TFA, cholesterol, vitamin D, and vitamin E were correlated with fatty acid proportions in adipose tissue. Full association results for all foods and nutrients are provided in the Supplementary material to allow interrogation of individual items (Tables S8–S10 and Figs. S3–S5).

### Adipose fatty acids and cardio-metabolic risk phenotypes

**Fig. 5:**
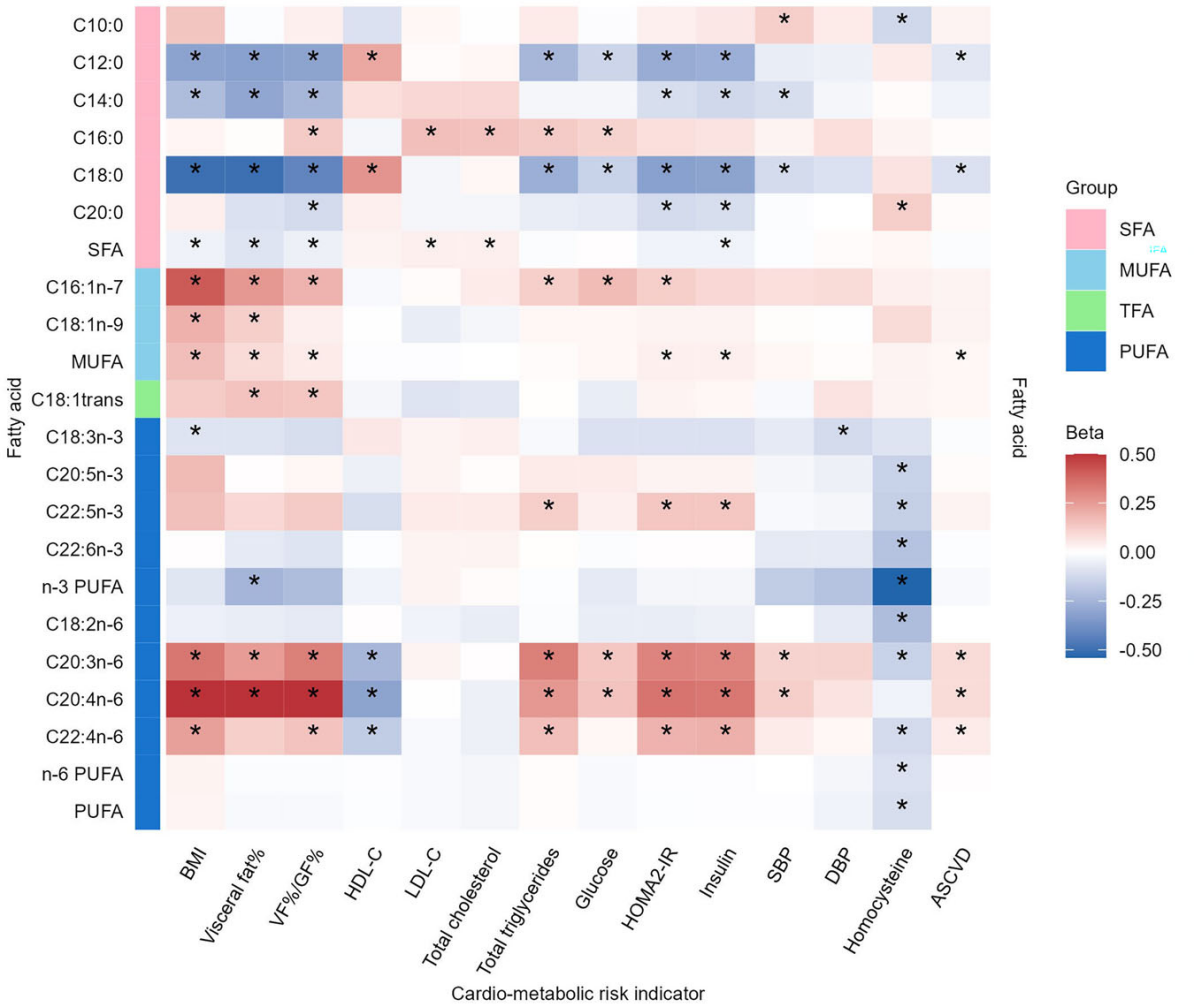
Associations between adipose fatty acids and cardio-metabolic risk phenotypes. The colour scale indicates the effect (Beta) of each fatty acid on cardio-metabolic risk phenotype. The asterisks show the significant (* *P*_FDR_ < 0.05). With the exception of BMI, the effects of the relationships between other indicators and fatty acids ranged from −0.5 to 0.5, so this figure rescaled the effect of BMI from −1.89 to 1.50 to −0.5 to 0.5. SFA saturated fatty acid, MUFA monounsaturated fatty acid, TFA trans-unsaturated fatty acid, PUFA polyunsaturated fatty acid, Visceral fat%, percentage of fat in the largest visceral fat region; VF%/GF%, percentage of fat in the largest visceral fat region/percentage of fat in the gynoid region, HDL-C or LCL-C high- or low-density lipoproteins cholesterol, SBP systolic blood pressure, DBP diastolic blood pressure, HOMA2-IR homoeostasis model assessment of insulin resistance, ASCVD Atherosclerotic Cardiovascular Disease risk score.

To identify the health effects of fatty acid accumulation in adipose tissue, we tested for the associations between adipose fatty acids and cardio-metabolic risk phenotypes. Overall, we identify extensive associations between adipose fatty acids and cardio-metabolic risk phenotypes, which are largely consistent across classes of fatty acids (saturated fatty acids vs unsaturated fatty acids) (Fig. 5). Visceral fat to gluteofemoral fat percentage ranked the highest number of associations with adipose fatty acids altogether, followed by BMI, and circulating levels of homocysteine and fasting insulin. Visceral fat percentage, visceral fat to gluteofemoral fat percentage and BMI were negatively associated with adipose SFAs proportions whereas were positively associated with MUFAs and n-6 PUFAs proportions, with the largest correlation magnitude of BMI (with coefficients [95% CI] ranging from −1.89 [−2.26 to −1.52] to −0.94 [−1.37 to −0.52] for SFAs and coefficients [95% CI] ranging from 0.47 [0.07 to 0.87] to 1.50 [1.10 to 1.89] for MUFAs and n-6 PUFAs, all *P*_FDR_ < 0.05) (Fig. 5 and Table S11). Glycemic indicators (HOMA2-insulin resistance, fasting insulin and fasting glucose) and total triglycerides showed a similar pattern. However, negative associations were found between plasma homocysteine level and adipose fatty acids, with coefficients [95% CI] ranging from −0.54 [−0.74 to −0.34] to −0.12 [−0.22 to −0.02] (all *P*_FDR_ < 0.05). HDL-C was positively correlated with two SFAs (C12:0 and C18:0, coefficient [95% CI] 0.22 [0.13 to 0.32] and 0.27 [0.17 to 0.36], respectively, both *P*_FDR_ < 0.05) but negatively correlated with three n-6 PUFAs (C20:3n-6, C20:4n-6, and C22:4n-6, with coefficients [95% CI] ranging from −0.31 [−0.41 to −0.20] to −0.17 [−0.27 to −0.08], all *P*_FDR_ < 0.05), in contrast to total triglycerides and ASCVD risk score. In summary, cardio-metabolic risk phenotypes were associated with adipose fatty acids proportions, where saturated and unsaturated fatty acids showed opposite directions, with the exception of C16:0 (palmitic acid) and C18:3n-3 (α-linolenic acid).

### Dietary patterns and cardio-metabolic risk phenotypes

Some habitual dietary patterns are known to lower the risk of cardio-metabolic diseases, and the associations between dietary patterns and cardio-metabolic biomarkers in TwinsUK has been reported previously (*N* = 2590) [29]. In order to confirm these associations were consistent within the subset of participants used in this study, the 427 twins with both diet and adipose data available, we validated the effect of adherence to dietary patterns on cardio-metabolic risk phenotypes in this study subset. Consistent with the previous TwinsUK results [29], we identified negative correlations between the dietary scores (AMED, DASH, HEI, AHEI and DQII) and visceral fat percentage or visceral fat to gluteofemoral fat percentage with coefficients [95% CI] ranging from −0.22 [−0.33 to −0.11] to −0.14 [−0.25 to −0.04] (all *P*_FDR_ < 0.05) (Table S12). We observed negative association between the HEI, AHEI and DQII scores and plasma homocysteine level with coefficients [95% CI] ranging from −0.17 [−0.27 to −0.07] to −0.13 [−0.23 to −0.03] (all *P*_FDR_ < 0.05).

### Dietary patterns, adipose fatty acids and cardio-metabolic risk phenotypes

**Fig. 6:**
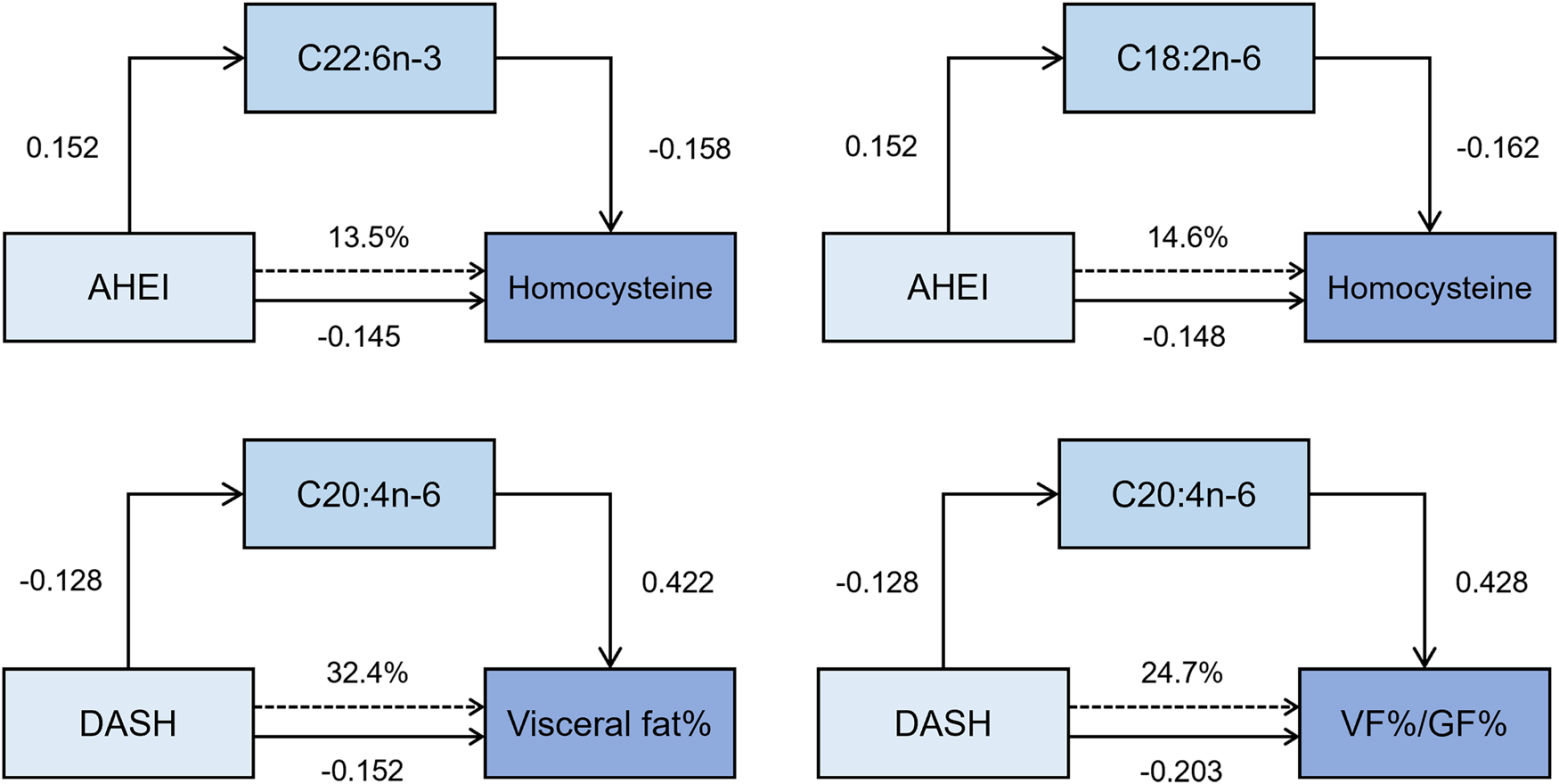
Direct effect and indirect mediation effect via the adipose fatty acid of dietary pattern affecting cardio-metabolic risk phenotype. The solid line shows direct association. The dotted line shows the total indirect effect, the number indicates the proportion of the effect of dietary pattern on the cardio-metabolic risk phenotype that goes through the putative mediator. For each association, the regression coefficient of the linear mixed-effects model is reported with significance (*P* < 0.05).

To investigate the putative mediation effects of adipose fatty acids on the associations between dietary scores and cardio-metabolic risk phenotypes, we selected 33 significantly associated triplets of dietary scores, adipose fatty acids, and cardio-metabolic risk phenotypes and performed mediation analyses (Table S13). Here, we included six dietary scores (AMED, DQII, HEI, AHEI, HDI and DASH), nine fatty acid proportions and four fatty acid sums (MUFA, n-3 PUFA, n-6 PUFA and PUFA), and four cardio-metabolic risk phenotypes (homocysteine, visceral fat percentage, visceral fat to gluteofemoral fat percentage, and systolic blood pressure). PUFAs, including n-3 PUFA C22:6n-3 (docosahexaenoic acid) and n-6 PUFA C18:2n-6 (linoleic acid), putatively mediated the negative association between AHEI and homocysteine level in plasma, with 13.5% and 14.6% of proportions mediated, respectively (Bonferroni-corrected *P* < 0.05) (Fig. 6). Similarly, PUFAs potentially mediated the effects of HEI on homocysteine level with higher mediation proportions (*P* < 0.05). We also identified suggestive results where C20:4n-6 (arachidonic acid) acted as potential partial mediators in the negative association between DASH and visceral fat percentage and the ratio of visceral fat to gluteofemoral fat percentage (proportion-mediated were 32.4% and 24.7%, respectively, *P* < 0.05).

In summary, we identified associations between CMD-related dietary patterns and adipose fatty acids, where HDI showed most associations. Multiple associations between CMD risk clinical phenotypes and adipose fatty acids were found, where saturated and unsaturated fatty acids showed opposite directions of effect. Adipose tissue arachidonic acid putatively mediated the association between DASH and visceral to gluteofemoral fat ratio.

### Sensitivity analysis

Participants in the study are all females aged from 39 to 85 years, and most experienced menopausal transition. Studies have documented distinct patterns of alterations in endogenous sex hormones and adverse changes in body fat distribution, lipids, lipoproteins, and vascular health over the menopause transition [41]. Seven fatty acid proportions in adipose tissue were different among three menopausal groups (pre-menopausal, post-menopausal without HRT, post-menopausal with HRT), commonly appearing between pre-menopausal and post-menopausal without HRT’ groups (Table S14). Therefore, we included menopausal groups as a covariate in sensitivity analysis (Model 2). We identified nine fatty acids in adipose tissue associated with BMI (Fig. 5), so we also considered BMI a covariate in sensitivity analysis (Model 3). Adipose tissue contains an array of cell types involving adipocyte, macrophage, and microvascular endothelial cells, whose proportions differ between samples within biopsies [42]. We found that half of the fatty acids in adipose tissue were associated with adipocytes and microvascular endothelial cells, and three fatty acids were associated with macrophages (Table S15). So, we included cell type proportions as well in sensitivity analysis (Model 4). In summary, our sensitivity analyses found no major changes to primary results when adjusting for additional covariates (Tables S7, S11 and S12). Mediation analyses based on the significant associations chosen from the analysis between dietary patterns, adipose fatty acids, and cardio-metabolic risk phenotypes of Model 2 (Model 1 + menopause status), Model 3 (Model 1 + BMI) and Model 4 (Model 1 + BMI + cell types) were performed as sensitivity analyses. Compared with the main analysis, no mediation effect of summary proportion of n-6 PUFA was found. However, the mediation effects of n-3 PUFA C22:6n-3 and n-6 PUFA C18:2n-6 were still robust after adjusting for additional covariates (proportion-mediated ranged from 13.3% to 17.8%, *P* < 0.05). Additionally, when adjusted for BMI, the mediation effect of C20:4n-6 was still robust (proportion-mediated were 19.3% and 21.2%, *P* < 0.05) (Table S13).

## Discussion

In this study, we identify broad associations between dietary patterns and fatty acid proportions in adipose tissue. We further find that adipose fatty acids are strongly associated with concurrently measured clinical cardio-metabolic risk phenotypes. We did not identify any association between adipose fatty acids and time of day or hours of fasting. In exploratory analyses, we identify potential fatty acid mediators of the relationship between dietary patterns and indicators of CMD. Moreover, we establish a resource reporting associations between dietary behaviours, nutrient intakes and food intakes and adipose tissue fatty acid proportions (Supplementary material).

Biological clocks are vital for health and are controlled by certain genes. However, continually shifting circadian factors can increase the risk of chronic diseases, including obesity, diabetes, cardiovascular diseases, cancer, and mood disorders [17]. Intermittent fasting and time-restricted diet have a wide range of effects on metabolic markers [43]. Studies suggested free fatty acid synthesis and degradation are under feeding-fasting and circadian regulation. ATP citrate lyase (ACLY), carnitine palmitoyl transferase (CPT) are rate-limiting enzymes for fatty acid synthesis and Beta oxidation. The circadian peak of ACLY expression coincides with feeding and the level of CPT also shows daily rhythms [18]. The circadian lipidomics study by Chua et al. has identified 35 rhythmically oscillating lipid species in human plasma. Notably, they reported that the majority of these circadian lipids were triacylglycerols and diacylglycerols, accumulating during the biological night and peaking around the habitual wake time [44]. However, in this study, fatty acid proportions in adipose tissue showed no association with clinical visit time and fasting hours, suggesting that adipose fatty acids are stable throughout the day, possibly due to the slow lipid turnover in human adipose tissue. This result contrasts with results from adipose tissue gene expression from the same individuals profiled here, a previous study identified the expression of 367 genes and 99 genes were associated with hours of fasting and time of day, respectively, and these genes were enriched for circadian rhythm processes [21].

Dietary intake can be reflected in fatty acid proportions in the adipose tissue over the years and has an important influence on fatty acid metabolism [45]. To our knowledge, no research linking dietary indices to fatty acid proportions in adipose tissue has been performed to date. In this study, we identified several associations between eight frequently reported dietary scores and adipose fatty acids. We identify several associations between HDI and adipose fatty acids, possibly due to the intake of SFA and PUFA from foods included for scoring HDI. The inverse relationship between HDI score and the energy proportions of SFAs from diet could explain the inverse link between HDI score and adipose proportions of SFAs [46]. Regarding other dietary patterns, the positive associations between healthy diet indexes and Mediterranean dietary scores and PUFAs have been documented in plasma [12, 47], in line with our study, suggesting that participants with high adherence to these dietary patterns may consume or generate more PUFAs. Linoleic acid is the most abundant PUFA of the eight tested PUFAs in adipose tissue (accounts for 12% of total fatty acids), and we observe the overall strongest associations between linoleic acid and dietary patterns among PUFAs. In addition, we identified negative associations between two PUFAs and DASH, and four other dietary patterns showed the same trend although with no significance due to the limited sample size.

Fatty acids in adipose tissue participate in a number of metabolic processes and are associated with cardio-metabolic health [48]. Epidemiology research has verified that higher eicosapentaenoic acid (EPA) and docosahexaenoic acid (DHA) proportions in plasma are protective for CMD [48], but are not related to T2D. In line with the previous results, our research observed negative correlations of EPA and DHA with homocysteine, but no significant associations with glycemic traits. N-6 PUFA proportions showed a negative association with HDL-C but positively correlated with adiposity (BMI, visceral fat percentage, and the ratio of visceral fat to gluteofemoral fat percentage), total triglycerides, and glycemic indicators, in line with previous plasma study [49, 50]. SFAs showed opposite directions compared to n-6 PUFAs in the current study, in line with previous studies [51]. Palmitic acid is the major SFA and the association of palmitic acid with total and LDL cholesterol concentration is consistent with experimental evidence demonstrating that this fatty acid raises these lipid concentrations [10]. Interestingly, we identified several fatty acids with significant associations to HDL-C and total triglycerides, but not to total cholesterol or LDL-C, possibly because much of the genetic regulation of HDL-C and total triglycerides occurs in adipose tissue, whereas LDL-C and total cholesterol are genetically regulated in the blood or liver [52].

Consistent with previous studies [53, 54], we confirmed that adherence to DASH and AMED diets was beneficially associated with CMD. These dietary scores were negatively associated with fat accumulation in the visceral area and homocysteine level in plasma. We also observed the favourable links between adipose fatty acids and the adherence to dietary scores designed based on healthy diet recommendations (HEI, AHEI and DQII).

Adipose fatty acids linked to both diet and cardio-metabolic disease risk phenotypes, for example, adipose PUFA proportions are associated with both (A)HEI, DASH and DQII and homocysteine and body fat composition. A goal of our study is to investigate how these three factors interact. We observed putative mediation effects of adipose PUFA proportions on the correlation between the HEI and homocysteine level in plasma, consistent with a previous study [55]. Given the evidence showing that adherence to DASH could reduce the risk of cardiovascular disease [53] and that arachidonic acid could potentially modify the risk of cardiovascular diseases [56–58], we hypothesised that arachidonic acid has a mediation role between DASH and cardio-metabolic risk phenotypes. The mediation analysis results were in line with this hypothesis. One possible explanation is that DASH diet has a favourable effect on insulin resistance with the consequence that less linoleic acid is converted to arachidonic acid, which is consistent with previous findings that the concentration of arachidonic acid in plasma and adipose tissue was associated with a lower risk of cardiovascular death [59].

Fatty acid profiles in adipose tissue reflect diet and are related to CMD risk factors. This study describes the relationships between dietary intake and adipose fatty acids and provides insights into their relationships with cardio-metabolic health. The results have several limitations. We only included females in the study, so the generalisability of the observations of this study is limited. The dietary intake was estimated utilising FFQ, which may introduce bias due to interviewee recall, incorrect portion estimation, and unavailability of up-to-date precise food composition database. The majority of FFQ-derived dietary scores in this study consider dietary fat intake of participants. In their scoring systems, OMED and AMED include MUFA/SFA, AHEI includes TFA, EPA and DHA, and PUFA, HEI includes PUFA + MUFA/SFA, DQII includes total fat, SFA, PUFA:MUFA:SFA, HDI includes SFA and PUFA. Although the type of fats participants used while cooking and baking were recorded in FFQ, the vegetable oil types which are important contributors to PUFA intake, are not considered in dietary scores. In addition, the associations shown in the study were based on a cross-sectional design and can not infer causality. In main analysis, we did not adjust for disease status and medication use, which may result in some residual confounders. Another limitation is that participants come to clinical visits in the daytime, thus circadian values only cover a portion of the day, and no samples were taken at night. We acknowledge that using time of visit and fasting hours as proxies for circadian factors represents a conceptual limitation, as these are not direct measures of circadian phase. Therefore, these analyses should be considered exploratory, and biological interpretations regarding circadian mechanisms warrant additional confirmation. The associations between dietary intake, adipose fatty acid proportions, and cardio-metabolic risk phenotypes may miss potential causal relationships. The mediation analysis in the current study only tested for one putative directionality, while other possible directionalities, interaction effects, or unmeasured confounders were not considered [60]. We tested a single mediator model but it is plausible that other unmeasured mechanisms also explain the relationship between exposure and outcome. Further longitudinal studies or randomised controlled trials conducted in the general population are needed, as these could potentially also provide sex-specific dietary recommendations for disease prevention.

In conclusion, this study demonstrates that adipose fatty acid composition represents dietary intake and is robustly associated with cardio-metabolic risk factors. Adipose fatty acid composition putatively mediates the links between dietary patterns and cardio-metabolic risk factors. Further research exploring the potential mechanisms is warranted.

## Supplementary information

TUK_Diet_AdiposeFattyAcid_CMD_SupplementalMethodsResultsFigures

TUK_Diet_AdiposeFattyAcid_CMD_SupplementalTables

## Data Availability

TwinsUK data are available to bona fide researchers upon application to the TwinsUK Resource Executive Committee (TREC). For information on how to apply, see https://twinsuk.ac.uk/researchers/access-data-and-samples/request-access/.
