# Supplementary material for: Dietary intake, fatty acid in adipose tissue and cardio-metabolic health: cross-sectional study from the TwinsUK cohort
Source: Nutr Diabetes. 2026 Jun 10;16:29. doi: 10.1038/s41387-026-00438-6 (PMC13478473; doi:10.1038/s41387-026-00438-6)
Supplement: Supplementary file 1 [file 41387_2026_438_MOESM1_ESM.docx]

**Supplementary Methods**

**Measures of cardio-metabolic risk phenotypes**

During the clinical visit, the height and weight of the participants were measured by trained nurses following the harmonized protocols to calculate BMI. Percentage of fat in the largest visceral fat region and percentage of fat in gynoid region were measured via dual-energy X-ray absorptiometry scans (Hologic QDR; Hologic, Inc.), and the ratio of these two was calculated afterward. The lipid profiles and glycaemic indicators levels were measured after an overnight fasting period according to standard procedures (Biochemistry Department, King’s College Hospital, London and Affinity Biomarker Labs, London, United Kingdom). Insulin resistance was calculated by the HOMA2 online application (https://www.dtu.ox.ac.uk/homacalculator/). Peripheral systolic blood pressure and diastolic blood pressure were measured after the participant took a seat and rested for at least three minutes using an automated cuff sphygmomanometer (OMRON HEM713C). The 10-year ASCVD risk score which is an algorithm utilized to estimate the cardiovascular risk of the participant. Detailed description of clinical indicators has been previously reported (1-3).

**Measurement of covariates**

The potential covariates included age, smoking status, physical activity, index of multiple deprivation (IMD), family relatedness, energy, alcohol intake, BMI, cell types, and menopausal status. Using self-reported lifestyle questionnaires targeting at specific aims, zygosity, smoking status, physical activity, socio-economic status, menopausal status and medications were also collected. Based on the postcode provided by the participants, a composite decile score IMD was calculated. IMD represents the area-level deprivation within the UK that includes seven domains: income deprivation; employment deprivation; health deprivation and disability; education, skills training; crime; barriers to housing and services; and living environment deprivation. It follows an established methodological framework in broadly defining deprivation to encompass a wide range of a participant’s living conditions. Daily alcohol (g/d) and energy intake (kcal/d) were estimated from the FFQ with FETA software. Menopausal status was categorized into three classes, pre-menopausal, post-menopausal with hormone replacement therapy (HRT), and post-menopausal without HRT based on questionnaires. Adipose tissue cell-type proportions involving adipocyte, macrophage, and microvascular endothelial cell composition were estimated from adipose tissue RNA-Seq data from TwinsUK using CIBERSORT, as previously described (4).

**Supplementary Results**

**Nutrient intake and adipose fatty acids**

People need many different nutrients to maintain health and reduce the risk of diet-related diseases. The amount of daily nutrient intake is calculated from FFQ and the mean and standard deviation of nutrient intake among 427 twins are described in Table S5. The mean macro-nutrient intakes of subjects were: fat 66.4 (SD 23.4) gram, carbohydrate 236.9 (SD 70.6) gram, and protein 82.2 (SD 22.0) gram. We aimed to explore associations between nutrient intake and adipose fatty acids, all results are shown in Fig. S3. Fat-related nutrients intakes comprising PUFA, SFA and TFA, cholesterol, vitamin D, and vitamin E were correlated with fatty acid proportions in adipose tissue. More specifically, participants’ consumptions of PUFA, cholesterol and vitamin E were positively correlated with adipose proportions of PUFAs (C18:2n-6, total PUFA and total n-6 PUFA) and PUFA/SFA but negatively correlated with adipose proportions of SFAs (C16:0 and total SFA). The consumptions of SFA and TFA were positively associated with adipose proportions of SFA (C20:0) and TFA (C18:1trans), respectively, but negatively associated with adipose proportions of n-3 PUFAs (C20:5n-3, C22:6n-3 and total n-3 PUFA). Moreover, the positive associations between the intakes of starch, non-starch polysaccharide (NSP), and maltose and adipose PUFAs proportions (C18:2n-6, total n-6 PUFA and total PUFA) and PUFA/SFA were highlighted, in contrast to their negative associations with SFA (C18:0 and total SFA).

We observed a broad range of positive associations between adipose proportion of C16:1n-7 and consumptions of carbohydrate, protein, vitamins and minerals. Besides adipose proportion of C16:1n-7, vitamin B family and vitamin C intakes were positively associated with C22:6n-3 proportion but negatively associated with C18:0 and n-6 PUFA/n-3 PUFA. Macro and micro mineral intakes were correlated with some adipose fatty acid proportions or ratios (C18:0, C16:1n-7, C18:1n-7, C22:6n-3, total PUFA, total n-6 PUFA, PUFA/SFA and n-6 PUFA/n-3 PUFA).

**Food intake and adipose fatty acids**

People consume a variety of food items every day to meet body requirement. The daily food intake of the participants is estimated from FFQ, and its characteristic is summarized in Table S6. To investigate whether certain food intake drives the associations, the associations between daily food intake (131 food items and 32 food groups) and fatty acids in adipose tissue were tested (Fig. S4 and Fig. S5). We observed most associations of adipose fatty acids with meat, dairy products, and fats. The intake of fresh red meat including lamb and beef was negatively correlated with adipose proportions of PUFAs (C18:2n-6, C22:6n-3, C20:3n-6, total PUFA, total n-3 PUFA and total n-6 PUFA). The intakes of dairy products including butter and cream were negatively correlated with PUFAs (C18:2n-6, C22:6n-3, total n-6 PUFA and total n-6 PUFA) and PUFA/SFA but margarine (polyunsaturated margarine) intake was positively correlated with those fatty acid proportions or ratio. The elevated butter intake was associated with increases in adipose proportions of SFAs, MUFA and TFA (total SFA, C14:0, C18:0, C16:1n-7 and C18:1trans). In addition, fish intake was associated with n-3 PUFAs in adipose tissue. The intake of fish fingers was positively associated with adipose proportion of C20:3n-6, and the intakes of white fish and oily fish were associated with adipose proportion of C22:6n-3. Interestingly, the intake of marrow was positively correlated with PUFAs proportions in adipose tissue.

**Sensitivity analysis**

To perform sensitivity analyses, we included menopausal status, BMI, and cell types in the regression model as well (Model 2-4). When adjusted for more covariates, no large alteration was observed (Table S8-S10).

**Supplementary Discussion**

Previous studies mainly investigated the relationship between dairy fat intake and omega-3 supplements and fatty acids in plasma or adipose tissue (5), the intake of other types of food and nutrients was neglected when estimating the dietary impact on fatty acid proportions. It is essential to employ comprehensive studies to explore further by incorporating dietary intake and fatty acid measures in adipose tissue. In the present study, we performed regression models to test the association between food and nutrient intake and fatty acid proportions in adipose tissue. We elucidated a positive correlation between fish intake and long-chain n-3 fatty acids (6, 7), as well as the consumption of n-6 PUFA-rich fish fingers (8) and adipose n-6 PUFAs proportions. Likewise, the intake of polyunsaturated margarine was positively associated with PUFAs proportions in adipose tissue. We also identified that the intakes of butter and cream were positively associated with SFAs in adipose tissue whereas fresh red meat and cream intakes were negatively associated with PUFAs. However, because of the various nutrient contents of food and *de novo* synthesis machinery of fatty acids, it is challenging to reveal the direct link between food intake and fatty acids.

Consistent with previous studies testing the association between dietary fatty acid intake and plasma phospholipid fatty acids and adipose-tissue-biopsy concentrations (9, 10), we identified associations between the intakes of fat-related nutrients comprising SFA, TFA, PUFA, cholesterol, and vitamin E and fatty acid proportions in adipose tissue but dietary intakes of PUFA and vitamin E showed opposite correlations compared with SFA, TFA, and cholesterol. Linoleic acid (LA) in adipose tissue has been generally accepted as a good indicator of dietary intake as its concentration in adipose tissue is primarily due to dietary intake. LA serves as the precursor of arachidonic acid and its metabolites and a natural ligand for PPARs, linked to lipid and glucose metabolism, immunology, obesity, and cardio-metabolic risk (11). In this present study, adipose LA was correlated with higher PUFA intake and lower intakes of cholesterol and SFA, although the exact amount of individual fatty acid intake is not available. TFA is an adipose tissue biomarker of TFA intake, in line with the previous study (12). What’s interesting is that we also identified an apparent linkage between carbohydrate and vitamin B consumption and adipose fatty acids, which may be due to excess carbohydrate transformation to fat stored in adipose tissue, and vitamin B acts as a coenzyme for fatty acid metabolism. Studies examined the correlations between vitamin B biomarkers and plasma fatty acids and pointed out that vitamin B intake was related to docosahexaenoic acid (DHA) and eicosapentaenoic acid (EPA) (13, 14).

**Supplementary References**

1.	Menni C, Migaud M, Glastonbury CA, Beaumont M, Nikolaou A, Small KS, et al. Metabolomic profiling to dissect the role of visceral fat in cardiometabolic health. Obesity (Silver Spring). 2016;24(6):1380-8.

2.	Menni C, Gudelj I, Macdonald-Dunlop E, Mangino M, Zierer J, Besic E, et al. Glycosylation Profile of Immunoglobulin G Is Cross-Sectionally Associated With Cardiovascular Disease Risk Score and Subclinical Atherosclerosis in Two Independent Cohorts. Circ Res. 2018;122(11):1555-64.

3.	Jennings A, MacGregor A, Welch A, Chowienczyk P, Spector T, Cassidy A. Amino Acid Intakes Are Inversely Associated with Arterial Stiffness and Central Blood Pressure in Women. J Nutr. 2015;145(9):2130-8.

4.	Glastonbury CA, Alves AC, Moustafa JSE, Small KS. Cell-Type Heterogeneity in Adipose Tissue Is Associated with Complex Traits and Reveals Disease-Relevant Cell-Specific eQTLs. Am J Hum Genet. 2019;104(6):1013-24.

5.	Hodson L, Skeaff CM, Fielding BA. Fatty acid composition of adipose tissue and blood in humans and its use as a biomarker of dietary intake. Prog Lipid Res. 2008;47(5):348-80.

6.	Lu Y, Zou L, Su J, Tai ES, Whitton C, Dam RMV, et al. Meat and Seafood Consumption in Relation to Plasma Metabolic Profiles in a Chinese Population: A Combined Untargeted and Targeted Metabolomics Study. Nutrients. 2017;9(7).

7.	Jacek M, Hrncirova D, Rambouskova J, Dlouhy P, Tuma P. Effect of Food with Low Enrichment of N-3 Fatty Acids in a Two-Month Diet on the Fatty Acid Content in the Plasma and Erythrocytes and on Cardiovascular Risk Markers in Healthy Young Men. Nutrients. 2020;12(8).

8.	Strobel C, Jahreis G, Kuhnt K. Survey of n-3 and n-6 polyunsaturated fatty acids in fish and fish products. Lipids Health Dis. 2012;11:144.

9.	Huybrechts I, Jacobs I, Aglago EK, Yammine S, Matta M, Schmidt JA, et al. Associations between Fatty Acid Intakes and Plasma Phospholipid Fatty Acid Concentrations in the European Prospective Investigation into Cancer and Nutrition. Nutrients. 2023;15(17).

10.	Cantwell MM. Assessment of individual fatty acid intake. Proc Nutr Soc. 2000;59(2):187-91.

11.	Guyenet SJ, Carlson SE. Increase in adipose tissue linoleic acid of US adults in the last half century. Adv Nutr. 2015;6(6):660-4.

12.	Baylin A, Kabagambe EK, Siles X, Campos H. Adipose tissue biomarkers of fatty acid intake. Am J Clin Nutr. 2002;76(4):750-7.

13.	Iglesia I, Huybrechts I, Gonzalez-Gross M, Mouratidou T, Santabarbara J, Chajes V, et al. Folate and vitamin B12 concentrations are associated with plasma DHA and EPA fatty acids in European adolescents: the Healthy Lifestyle in Europe by Nutrition in Adolescence (HELENA) study. Br J Nutr. 2017;117(1):124-33.

14.	Kim H, Enrione EB, Narayanan V, Li T, Campa A. Associations of Vitamin B6 Intake and Plasma Pyridoxal 5'-Phosphate with Plasma Polyunsaturated Fatty Acids in US Older Adults: Findings from NHANES 2003-2004. Nutrients. 2022;14(11).

**Supplementary Figures**

**Fig. S1:** Correlation among dietary scores. The colour scale indicates the Pearson correlation coefficient between dietary scores and the colour intensity represents the degree of the coefficient. OMED, Original Mediterranean Score; AMED, Amended Mediterranean Score; DASH, Dietary Approaches to Stop Hypertension; NDS, Nordic Dietary score; HEI, Healthy Eating Index; AHEI, Alternative Healthy Eating Index; HDI, Healthy Diet Indicator; DQII, Diet Quality Index International.

**Fig. S2**: Violin plots of cardio-metabolic risk phenotypes. SBP, systolic blood pressure; DBP, diastolic blood pressure; HDL-C, high-density lipoproteins cholesterol; LDL-C, low-density lipoproteins cholesterol; HOMA2-IR, homeostasis model assessment of insulin resistance; Visceral fat%, percentage of fat in the largest visceral fat region; VF%/GF%, the ratio of percentage of fat in the largest visceral fat region to percentage of fat in gynoid region; ASCVD, Atherosclerotic Cardiovascular Disease risk score.

**Fig. S3:** Associations between nutrient intake and adipose fatty acids. The colour scale indicates the effect (β) of each nutrient on fatty acid. The asterisks show the significant (* *P_FDR_* < 0.05). NSP: non-starch polysaccharides; SFA, saturated fatty acid; MUFA, monounsaturated fatty acid; TFA, trans-unsaturated fatty acid; PUFA, polyunsaturated fatty acid.

**Fig. S4:** Associations between food group intake and adipose fatty acids. The colour scale indicates the effect (β) of each food group on fatty acid. The asterisks show the significant (* *P_FDR_* < 0.05). SFA, saturated fatty acid; MUFA, monounsaturated fatty acid; TFA, trans-unsaturated fatty acid; PUFA, polyunsaturated fatty acid.

**Fig. S5**: Associations between food intake and adipose fatty acids. The colour scale indicates the effect (β) of each food on fatty acid. The asterisks show the significant (* *P_FDR_* < 0.05). SFA, saturated fatty acid; MUFA, monounsaturated fatty acid; PUFA, polyunsaturated fatty acid.
